# Huang-Lian-Jie-Du-Tang Protects Rats from Cardiac Damages Induced by Metabolic Disorder by Improving Inflammation-Mediated Insulin Resistance

**DOI:** 10.1371/journal.pone.0067530

**Published:** 2013-06-28

**Authors:** Chuan Bao Li, Xiao Xing Li, Yu Guo Chen, Hai Qing Gao, Pei Li Bu, Yun Zhang, Xiao Ping Ji

**Affiliations:** 1 The Key Laboratory of Cardiovascular Remodeling and Function Research, Chinese Ministry of Education and Chinese Ministry of Public Health, Shandong University Qilu Hospital, Jinan, Shandong, China; 2 Department of Emergency, Shandong University Qilu Hospital, Jinan, Shandong, China; 3 Department of Geriatrics, Shandong University Qilu Hospital, Jinan, Shandong, China; Pennington Biomedical Research Center, United States of America

## Abstract

Huang-lian-jie-du-tang (HLJDT), a traditional Chinese medicine, has been shown to improve insulin resistance (IR) induced by inflammation, a key event in the development of metabolic syndrome (MS). The present study aimed to investigate the protective effects of HLJDT on MS and explore the underlying mechanism. MS rats were established with obese-diets and treated with normal saline, aspirin or HLJDT. The myocardial lesions were identified by echocardiogram, transmission electron microscope, and Sirius-red staining. The inflammatory cytokines were measured by ELISA and real-time PCR. The activation of NF-κB, JNK, SOCS3, IRS1 and AKT in the heart was detected by immunohistochemistry and Western blot analysis. Compared with the controls, MS rats developed obvious obesity, hypertension, dyslipidemia, IR, inflammation, and cardiac damage. Moreover, phosphorylated IRS-1 at Ser307 was correlated with the activation of NF-κB, JNK and SOCS3 and the inhibition of AKT in the heart from MS rats. These data suggest that serine phosphorylation of IRS-1 in response to inflammation is mediated, in part, by NF-κB, JNK and SOCS3. Notably, HLJDT inhibited the activation of NF-κB and reduced serine phosphorylation of IRS-1. In summary, HLJDT protects myocardium from IR-mediated injury by inhibiting serine phosphorylation of IRS-1 in MS rats.

## Introduction

The constellation of metabolic abnormalities including centrally distributed obesity, decreased high-density lipoprotein cholesterol (HDL-C), elevated blood pressure (BP), and hyperglycaemia is known as the metabolic syndrome (MS). MS is very common in the population and presents a precursor state for cardiovascular disease [1]. Central obesity and insulin resistance (IR), two main disorders of the syndrome, are important risk factors for cardiovascular disease [2]. Moreover, anti-inflammatory and proinflammatory molecules such as TNF-α, IL-1 and IL-6 play an important role in IR [3], [4]. A key mechanism by which inflammatory cytokines induce IR involves serine phosphorylation of insulin receptor substrate (IRS)-1 [5]–[7]. IRS-1 is phosphorylated at serine sites by several pathways, including IKK/NF-κB (IκB kinase/nuclear factor-κB), JNK (c-jun N-terminal kinase), and SOCS (suppressors of cytokine signaling) [8]–[10]. Therefore, the strength of insulin signaling is reduced via the IRS-1/phosphatidylinositol (PI) 3-kinase pathway, resulting in diminished metabolism of glucose and fat in insulin target tissues, such as liver, skeletal muscles and adipose tissue.

Huang-lian-jie-du-tang (HLJDT) is an important remedy in traditional Chinese medicine and has been used for the treatment of hypertension, apoplexia and palpitation. In addition, HLJDT can improve chronic inflammatory disease such as rheumatoid arthritis [11]. Although anti-inflammatory effect of HLJDT is definite, very few studies have investigated the mechanisms by which HLJDT improves inflammation-mediated IR in MS.

In this study, we investigated the potential role of inflammatory pathways such as IKKβ, JNK and SOCS3 in the insulin-signaling cascade in the hearts from MS rats. We also explored the molecular mechanisms by which HLJDT improves IR and protect myocardium from myocardial remodeling. Aspirin has been shown to improve IR and myocardial function [12]–[14]. Therefore, we included aspirin treatment as a baseline to evaluate the efficacy of HLJDT on IR in MS.

## Materials and Methods

### Composition and Preparation of Huang-Lian-Jie-Du-Tang (HLJDT)

HLJDT were purchased from Jianlian Company of Traditional Crude Drugs (Jinan, China), and carefully authenticated by Dr. Xiang-Hong Liu (Pharmaceutical Preparation Section, Shandong University Qilu Hospital, Jinan, China). Voucher specimens (numbers were listed in Table 1) were deposited at the Herbarium of Shandong University (Jinan, China). Rhizoma coptidis, Radix scutellariae, Cortex phellodendri and Fructus gardeniae were mixed in the ratio of 3∶2:2∶3 and the total weight was 500 g. The mixture was decocted twice by refluxing with water (1∶10 and then 1∶5 w/v) for 1 h, and the solution obtained was concentrated to give an extract of 330 g. The yield of HLJDT extract was about 66.7% (w/w) according to the original herbs and the whole processes were manipulated by Pharmaceutical Preparation Section, Shandong University Qilu Hospital, Jinan, China. For the extract, the content of berberine hydrochloride was 18 mg/g measured by the lamellar scanning method. The resulting products were stored at 4°C and diluted to the desired concentrations with PBS (pH 7.4) and filtered before use.

**Table 1:** Recipe of Huang-Lian-Jie-Du-Tang (HLJDT) formulation.

Components	Voucher specimens number	Part used	Amount used (g)
Coptis chinensis Franch	SDU0186	Root	150
Scutellaria baicalensis Georgi	SDU0174	Root	100
Phellodendron amurense Rupr	SDU0165	Bark	100
Gardenia jasminoides Ellis	SDU0201	Fruit	150

### Animals

The animal studies were approved by the Animal Ethics Committee of Shandong University. Six-week-old 130–150 g Wister male rats (n = 75) were obtained from Shandong University (Jinan, China). Rats were randomized into two diet groups: one group (*n* = 10) was fed normal diet and the other group (*n* = 65) was fed with obese-diet (2% fat, 10% sucrose, 6% salt and 8% defatted milk powder )and drinking water (20% sucrose solution) at libitum. After 16 weeks, 24-h fasting blood samples, final body weight (BW) and systolic blood pressure (SBP) were obtained. Compared with the controls, the obese-fed rats with MS were identified, using criteria analogous to ATP III as ≥3 of the following: hypertriglyceridemia, low HDL-C, high fasting glucose, excessive waist circumference, and hypertension. 32 MS rats were randomly divided into three groups: MS+HLJDT group (MS+H, n = 11), MS+Aspirin group (MS+A, n = 11), and MS group (n = 10). All of the rats were fed obese diet before and after treatment, and dosed orally with HLJDT (1.04 g/100 g), aspirin (120 mg/kg), and equal volume of PBS, respectively, every day for 12 weeks. Age matched control Wistar rats (n = 10) were randomly selected (NC group), which were fed normal diet throughout the experiments and dosed orally with equal volume of PBS every day for 12 weeks. HLJDT was dissolved in PBS and the rats were monitored to ensure HLJDT was completely consumed.

### Analysis of BW, SBP and Heart Rate

BW was recorded every two weeks in the morning throughout the experiment, together with SBP and Heart Rate (HR) by the tail-cuff method using a Rat Tail Manometer provided by Japanese and Chinese Friendly Hospital (RBT-1; Beijing, China).

### Analysis of Fasting Insulin, Glucose and Lipid Profiles in the Serum

Samples of venous blood were collected, fasting insulin (FINS), glucose (FBG), and lipid profiles (triglyceride, cholesterol, HDL-C, LDL-C) were measured after 12-hour fasting every two weeks. Insulin resistance index was calculated as follows: IRI = (FBG*FINS)/22.5.

### Echocardiogram Analysis

Echocardiogram was performed on left ventricular (LV) at baseline (6 week), after 16 week, and at the end of the study, using Hewlett-Packard equipment, Sonos 7500 model, and an electronic 10-MHz transducer. All the animals were anesthetized with 10% chloral hydrate (0.3 ml/100 g) by intraperitoneal injection, and, after epilation of the abterior region of the thorax, they were positioned in the left lateral decubitus position. Two-dimensional and M-mode images from the parasternal long and short axis were obtained and recorded on videotape for later review. The cardiac structures were measured in at least three consecutive heart cycles. The left ventricle diastolic and systolic diameter (LVDd and LVSd), interventricular septum (IVS), and the left ventricle posterior wall thickness (LVPW) were measured. The left ventricular fractional shortening (FS) was calculated using the following equation: FS (%) = [(LVDd – LVSd)/LVDd] × 100%. Pulse-wave Doppler spectra (E and A wave velocity) of mitral inflow were recorded from the apical 4-chamber view, with the sample volume placed near the tips of the mitral leaflets. The peak velocities in the rapid ventricular lilling phase (E wave) and during atrial contraction (A wave) were measured from the videotape in 3 consecutive cardiac cycles.

### Sirius Red Staining

At the end of the experiment, the rats were executed. Then, the heart was isolated at the aortic root and both atria were removed. A part of LV was cut transversly parallel to the atrio-ventricular groove at the equatorial plane and embedded in paraffin, then 5 µm-thick sections of LV were cut and stained with the collagen-specific Sirius red for the measurement of the interstitial fibrosis.

### Immunohistochemical Staining

The sections were dewaxed and rehydrated, then soaked in 1% citric acid buffer, pH 6.0 at 92–98°C for 15 min for antigen retrieval. The slides were cooled at room temperature for 30 min, rinsed with PBS, and incubated in 5% bovine serum albumin for 20 min to block nonspecific binding. The slides were incubated with 1∶200 rabbit polyclonal anti-rat NF-κB p65 antibody (Abcam, USA) or 1∶200 rabbit polyclonal anti-rat ICAM-1 antibody (Santa Cruz Biotechnology, USA) overnight at 4°C, then incubated with biotinylated anti-rabbit IgG secondary antibodies. DAB substrate kits (Vector Labs) were used to reveal the immunohistochemical reaction. PBS was used instead of primary antibody as a negative control.

### Transmission Electron Microscope (TEM)

A part of the myocardium, about 0.5×1×5 mm^3^, was fixed with 2.0% glutaraldehyde for electron microscopic examination. The tissues were examined by using an H-7000FA transmission electron microscope (Hitachi Co. Ltd., Tokyo, Japan) for ultrastructural changes in cardiomyocytes.

### Quantitative Reverse Transcription–polymerase Chain Reaction (qRT–PCR)

Total RNA was prepared from myocardium samples using Trizol reagent (Invitrogen, Inc., Carlsbad, CA, USA), according to the manufacturer’s instructions. Total RNA was treated with DNase (DNAfreeTM, Ambion Inc., Austin, TX, USA) to remove genomic DNA and quantified using a spectrophotometer (Eppendorf Co. German). Three microgram of total RNA was reverse transcribed into cDNA using Murine Moloney Leukemia virus (M-MLV) reverse transcriptase (Promega, USA). qRT–PCR was performed using SYBR Green reagent (TaKaRa Corp., Kyoto, Japan) and primers specific for collagen I/III, TGF-β1, IL-6, TNF-α and ICAM-1 mRNA on an Lightcycler (Roche applied science, Germany). Primer sequences and optimal PCR annealing temperatures and cycle numbers were listed in Table 2, and transcript levels were normalized to β-actin, which was used as an endogenous control. The 2^–ΔΔCt^ relative quantification method was used to calculate fold difference in transcript levels between samples.

**Table 2:** Primer sequences used in this study.

Gene	rimer sequence(5′ –3′)	Product length (bp)	Annealing temp(°C)	Cycle
IL-6	GTCAACTCCATCTGCCCTTC	164	60	40
	ACTGGTCTGTTGTGGGTGGT			
ICAM-1	TTTCGATCTTCCGACTAGGG	112	60	40
	AGCTTCAGAGGCAGGAAACA			
TNF-α	AGTCCGGGCAGGTCTACTTT	175	60	40
	TGAGCCACAATTCCCTTTCT			
TGFβ1	GGTCACTTTCACTGGTTGACGA	106	55	40
	TTGAATATCAAACACGCAAGGC			
Collagen I	TTCACCTACAGCACGCTTGT	196	57	40
	TTGGGATGGAGGGAGTTTAC			
Collagen III	GGTCACTTTCACTGGTTGACGA	201	55	40
	TTGAATATCAAACACGCAAGGC			
β-actin	CGTTGACATCCGTAAAGACC	176	60	40
	TAGAGCCACCAATCCACACA			

IL-6: interleukin-6; ICAM-1: intercellular adhesion molecule-1; TNF-α: tumor necrosis factor-alpha; TGFβ1: transforming growth factor-beta 1; collagen I/III: collagen types I/III; IKK: IκB kinase.

### Enzyme-linked Immunosorbent Assay (ELISA)

Serum samples were collected from the rats every two weeks and kept frozen at -80°C until analysis of TNF-α level by a commercially available ELISA kit (Bender MedSystem, Austria, Vienna) according to the manufacturer’s instructions. The reaction was terminated by the addition of acid and the absorbance was measured at 450 nm. A standard curve was prepared from seven standard dilutions and serum TNF-α level was determined.

### Western Blot Analysis

The frozen LV tissues were extracted and equal amounts of protein (50 µg) were fractionated on 10% SDS-polyacrylamide gels and then transferred to nitrocellulose membranes. Membranes were blocked at 4°C with 5% nonfat milk in Tris-buffered saline (25 mM Tris, 137 mM NaCl, and 2.7 mM KCl) containing 0.05% Tween-20 and then incubated overnight at 4°C with the following primary antibodies: rabbit polyclonal anti-rat NF-κB antibody (Abcam, USA, dilution: 1∶500), rabbit polyclonal anti-rat SOCS3 antibody (Abcam, USA; dilution: 1∶400), rabbit polyclonal anti-rat phospho-JNK (Thr183/Tyr185) antibody (Cell Signaling Technology, USA; dilution: 1∶1000), rabbit polyclonal anti-rat phospho-IRS-1 (Ser 307) antibody (Cell Signaling Technology, USA; dilution: 1∶1000), rabbit polyclonal anti-rat phospho-Akt (Ser473) antibody (Cell Signaling Technology, USA; dilution: 1∶1000) or β-actin (Santa Cruz, USA; dilution: 1∶1000). Then the membranes were washed three times in TBS-T and incubated with horseradish peroxidase-conjugated secondary antibody at room temperature. Immunoreactive bands were visualized using an enhanced chemoluminescence and quantified by image analyzer (AlphaImager 2200, USA).

### Statistical Analysis

Data were expressed as the mean±S.D. One-way ANOVA followed by a Tukey–Kramer post-hoc test was used for statistical comparison among the various treatment groups. Unpaired *t*-test was used to compare data between obese fat-fed rats and normal-fed rats. *P*<0.05 was considered statistically significant.

## Results

### HLJDT Affects Baseline Measurements for BW, SBP and HR

**Figure 1:**
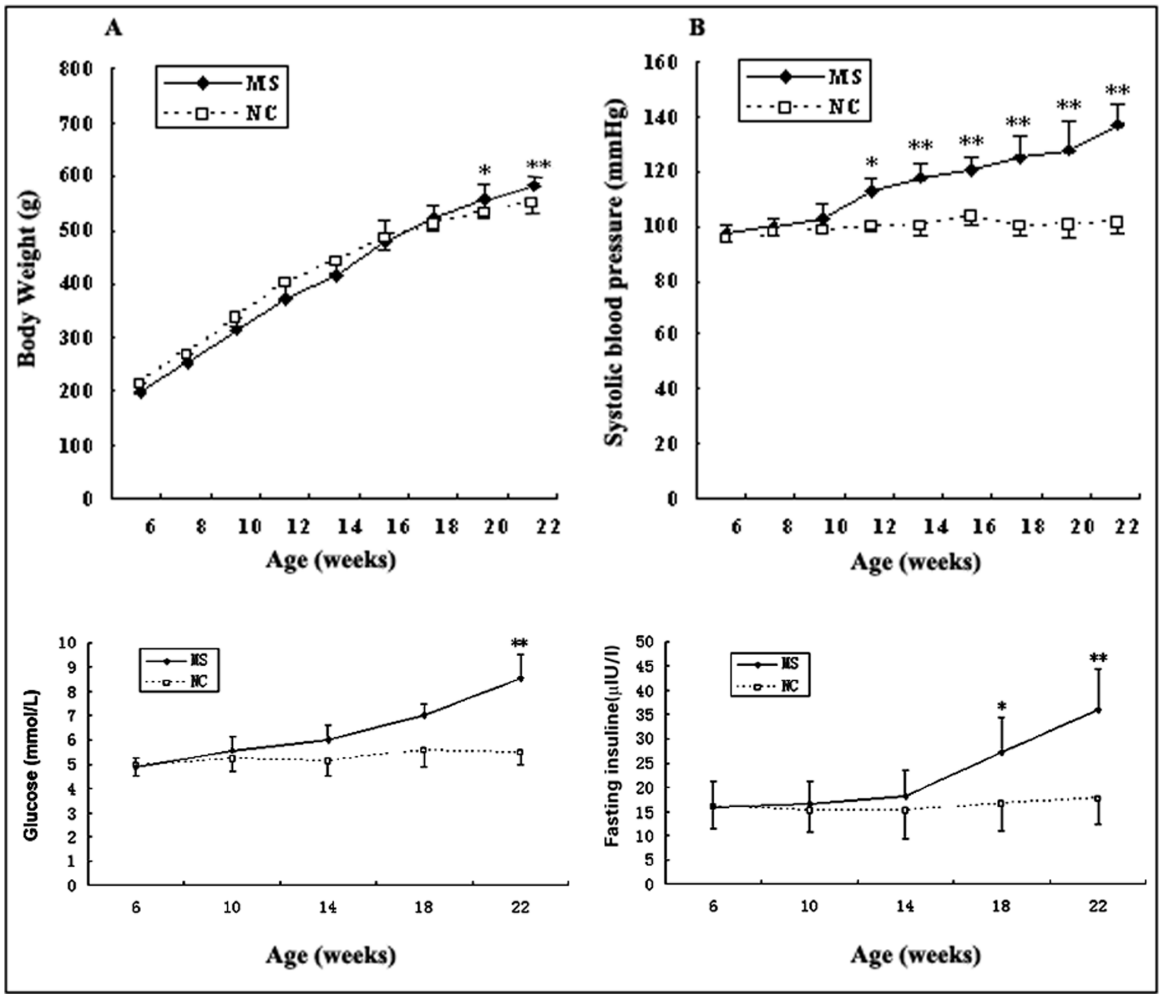
Body weight, systolic blood pressure, fasting blood glucose and fasting insuline were measured from 6 weeks to 22 weeks. Note a marked increase of body weight, systolic blood pressure, fasting blood glucose and fasting insuline from 20 wk, 12 wk, 22 wk and 18 wk, respectively. **P<0.01 vs. the normal-fed rats, *P<0.05 vs. the normal-fed rats.

At baseline, mean BW and SBP were similar between the normal-fed and obese-fed rats (P>0.05). After 16 weeks, the obese-fed rats were significantly overweight and had higher SBP (7% and 36% increase, respectively) compared with the normal-fed rats (P<0.05) (Fig. 1). After 12-week treatment with aspirin or HLJDT, BW was markedly reduced by 18% or 17%, respectively (Table 3). SBP was reduced by 8% in HLJDT-treated rats but not significantly changed in aspirin-treated rats (Table 3). There was no significant difference in HR among all groups (Table 3).

**Table 3:** General characteristics in the four groups after administration.

	NC (n = 10)	MS (n = 10)	MS+A (n = 11)	MS+H (n = 11)
BW	565.58±26.27	662.87±25.79**	545.98±34.87##	554.29±35.26##
SBP	101.83±4.45	136.89±7.02**	133.51±7.13	126.43±7.52##
HR	412.89±38.65	407.69±53.82	408.58±40.79	419.78±45.39
FBG	5.29±0.52	8.72±0.96**	8.11±0.79	6.59±0.78##
FINS	17.89±5.36	36.15±9.86**	25.85±7.88#	24.89±8.46#
IRI	5.28±1.31	13.96±3.19**	8.05±2.16#	6.89±2.12##
TG	0.75±0.27	0.92±0.25	0.75±0.18	0.74±0.36
HDL-C	1.38±0.21	1.06±0.15**	1.19±0.20	1.35±0.21#
LDL-C	0.36±0.06	0.40±0.06	0.37±0.05	0.36±0.05

All data were mean±SD. BW (g): body weight; SBP: blood pressure (mmHg); HR (beats/minute): heart rate; FBG (mmol/L): fasting blood glucose; FINS (µIU/l): fasting insuline; IRI: insulin resistance index; TG (mmol/L): triglyeride; HDL-C (mmol/L): HDL-cholesterol; LDL-C (mmol/L): LDL-cholesterol.

** *P*<0.01,

* *P*<0.05 vs. NC group;

## *P*<0.01,

# *P*<0.05 vs. MS group.

### HLJDT Affects Baseline Measurements for Serum Values

At baseline, all the measured values, including FBG, FINS, TG, CHO, HDL-C and LDL-C, were similar between the normal-fed and obese-fed rats (P>0.05). 16 weeks later, the obese-fed rats tended to have higher CHO, FBG and FINS levels (21%, 69% and 54% increase, respectively), and lower HDL-C (17% decrease) compared with the normal-fed rats (P<0.05). At the end of the experiment, MS rats had the greatest decrease in HDL-C levels and increase in FINS and FBG levels compared with NC group (Table 3). Furthermore, there was significant decrease of FINS level in both aspirin and HLJDT-treated groups compared with MS group (Table 3). HDL-C levels were increased by 11% and 22% in aspirin-treated and HLJDT-treated rats, respectively (Table 3).

### HLJDT Improves Cardiac Function

**Figure 2:**
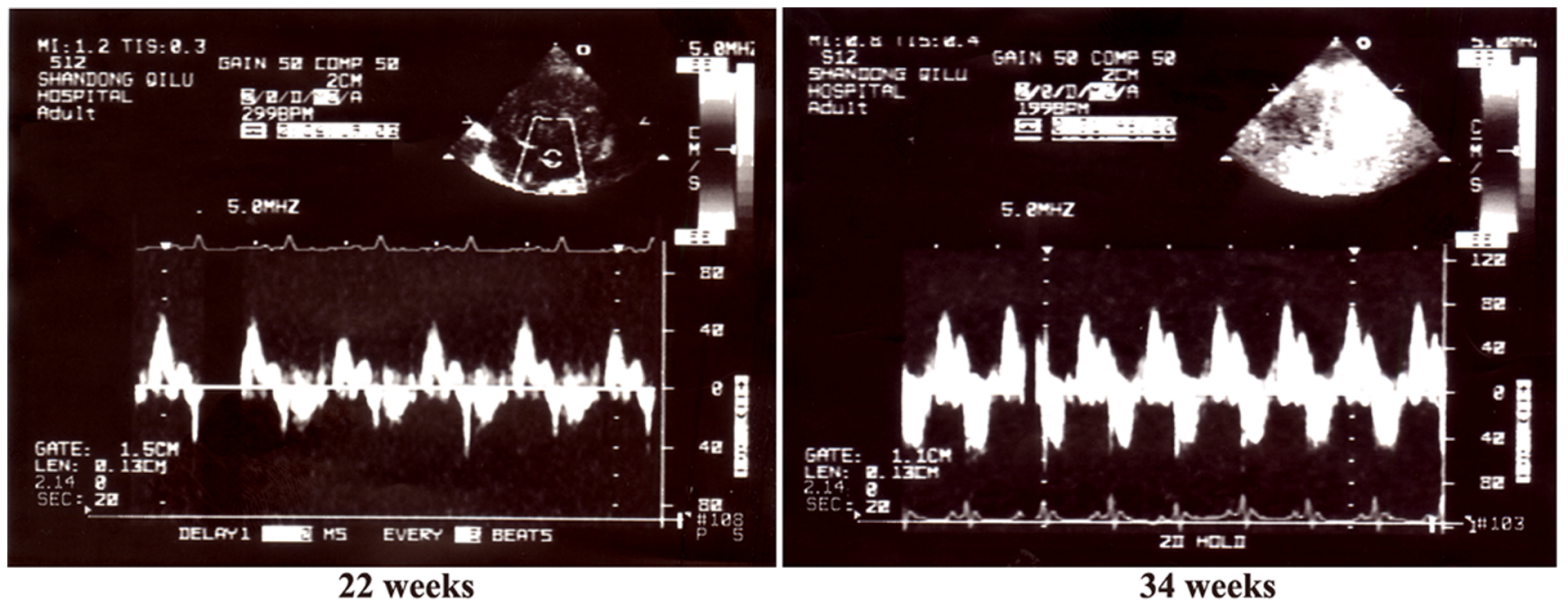
Transmitral inflow patterns (E and A wave) for MS rat at 22 and 34 weeks. Note increased A waves and decreased E/A in the MS group.

Characteristics of echocardiogram at baseline showed no significant differences between the normal-fed and obese-fed rats (P>0.05). After 16 weeks, IVS and LVPW in the obese-fed rats obviously increased (9% and 9% compared with the controls, respectively), indicating the presence of LV concentric hypertrophy (P<0.05). FS, representing LV systolic function, was not different between the obesity-fed and normal-fed rats (P>0.05). Furthermore, E/A was decreased by 23% in the MS rats (P<0.05) (Fig. 2). At the end of the study, there were more marked changes in IVS, LVPW and E/A (Table 4). Additionally, compared with the MS rats, treatment with either aspirin or HLJDT significantly slowed the progression of cardiac hypertrophy and protected the diastolic function, as estimated by IVS, LVPW and E/A (Table 4). Moreover, there were no significant differences between aspirin-treated and HLJDT-treated groups.

**Table 4:** Parameters measured by echocardiogram.

	NC(n = 10)	MS(n = 10)	MS+A(n = 11)	MS+H(n = 11)
LVDd	5.36±0.39	5.43±0.42	5.39±0.43	5.33±0.45
LVSd	2.19±0.19	2.33±0.26	2.23±0.24	2.09±0.23
IVS	1.73±0.06	2.16±0.17**	1.96±0.13##	1.89±0.12##
LVPW	1.86±0.11	2.19±0.12**	1.99±0.06##	1.93±0.07##
FS	0.59±0.02	0.58±0.05	0.57±0.05	0.59±0.04
A wave	35.89±8.47	64.76±12.76**	51.67±10.35#	52.36±11.79#
E/A	2.13±0.19	1.36±0.26**	1.67±0.23#	1.69±0.22#

All data were mean±SD. LVDd (mm): left ventricular end-diastolic dimension; LVSd (mm): left ventricular end-systolic dimension; IVS (mm): intraventricular septal wall; LVPW (mm): left ventricular posterior wall thickness; FS: fractional shortening; A wave (cm/s): peak late diastolic flow velocity, E/A: ratio of peak early diastolic filling velocity to peak velocity at atrial contrations.

** *P*<0.01,

* *P*<0.05 vs. NC group;

## *P*<0.01,

# *P*<0.05 vs. MS group.

### HLJDT Improves Ultrastructure of Cardiomyocytes

**Figure 3:**
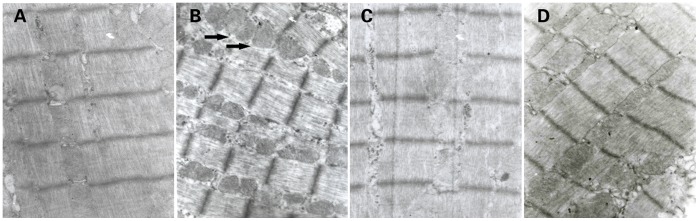
Cardiac lesions measured by transmission electron microscopy. A: NC group (n = 10). Note the regular membrane and intercalated disc between adjacent myocytes. B: MS group (n = 10). Interrupted capillary membrane, and derangement and increase of myofibers and mitochondria in cardiomyocytes were visible. Swollen mitochondria were shown by the arrows. C: MS+A group (n = 11). D: MS+H group (n = 11). Compared with MS group, the ultrastructural changes of drug-fed groups were obviously improved. Original magnification: ×10, 000.

TEM analysis showed the derangement of myofibers, and swollen mitochondria in cardiomyocytes in MS rats compared with the NC rats (Fig. 3). Moreover, after aspirin or HLJDT treatment, the cardiac ultrastructure was obviously improved (Fig. 3).

### HLJDT Affects Collagen Contents

**Figure 4:**
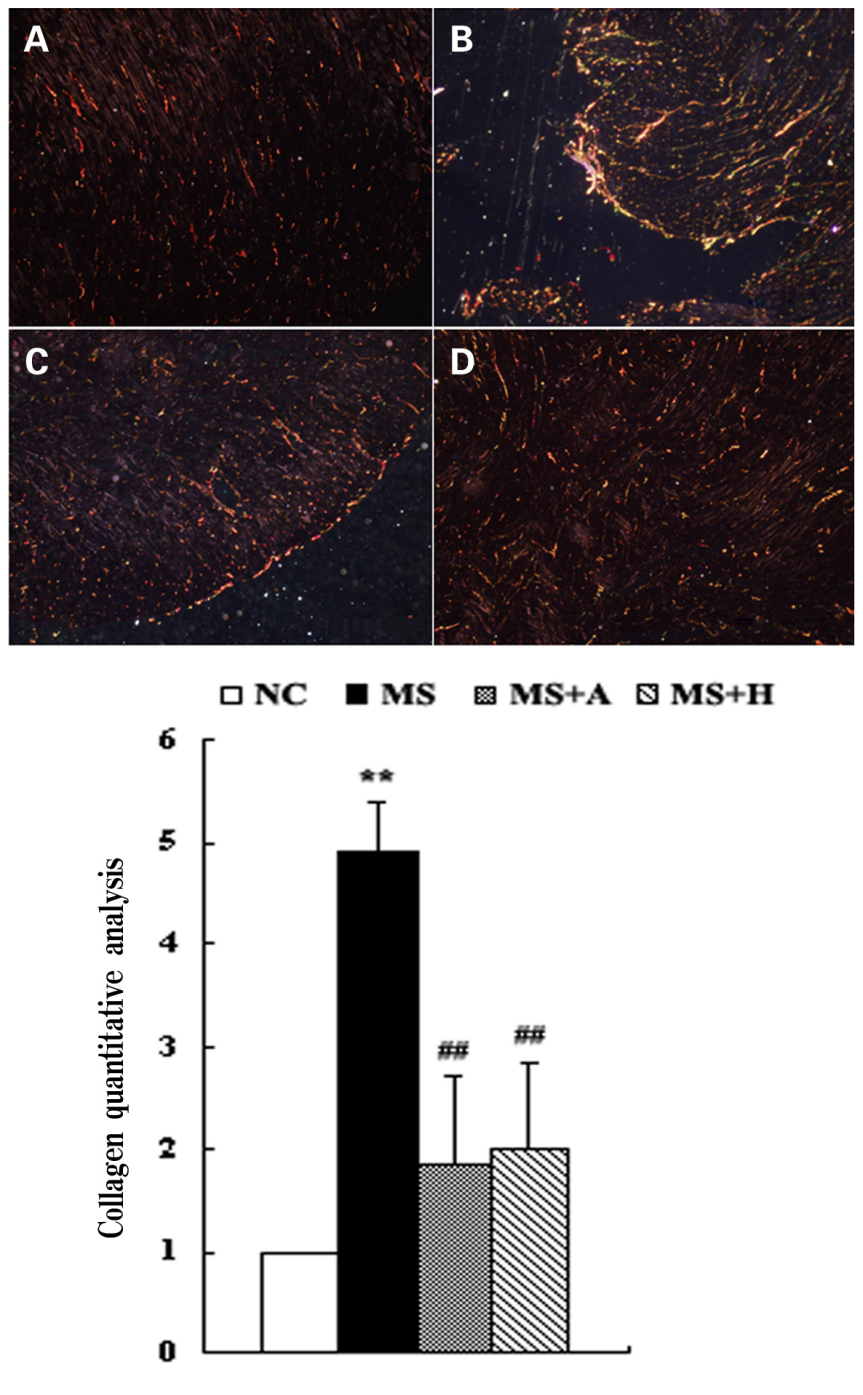
Cardiac fibrosis measured by Sirius Red staining. The top panels showed representative images of Sirius Red-stained LV sections. A: NC group (n = 10); B: MS group (n = 10); C: MS+A group (n = 11); D: MS+H group (n = 11). The bottom bar graph showed quantitative interstitial fibrosis ratio. Each bar represented the mean ±SD. **P<0.01, *P<0.05 vs. NC; ^##^P<0.01, ^#^P<0.05 vs MS. Original magnification, ×400.

Sirius red staining revealed an increase of interstitial fibrosis in the myocardium of MS rats compared with the NC group. Furthermore, a fraction of interstitial fibrosis was prevented by both aspirin and HLJDT (Fig. 4).

### HLJDT Decreases Serum TNF-α Level

**Figure 5:**
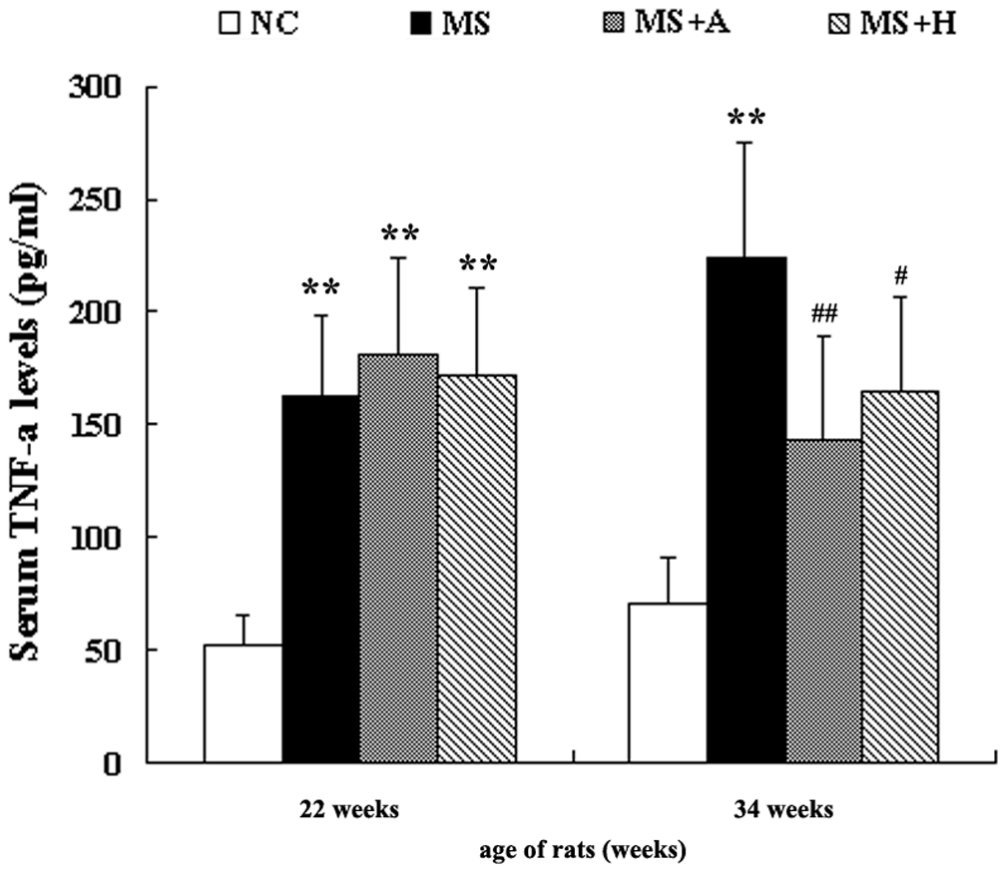
Serum concentration of TNF-α measured by ELISA. The left bar graph indicated serum concentration of inflammatory cytokines before administration, while the right one indicated serum concentration of inflammatory cytokines after the administration of aspirin or HLJDT for 12 weeks. Each bar represented the mean ±SD. **P<0.01, *P<0.05 vs. NC; ^##^P<0.01, ^#^P<0.05 vs. MS. NC group (n = 10); MS group (n = 10); MS+A group (n = 11); MS+H group (n = 11).

At baseline, the mean value of TNF-α was similar in the obese-fed and normal-fed rats. Serum TNF-α level of MS rats was 4 fold higher than in the controls at 16 weeks (Fig. 5). The difference persisted till the end of the study. However, the elevated TNF-α level was decreased by 36% in aspirin group and 26% in HLJDT group (Fig. 5).

### HLJDT Decreases NF-κB p65 and ICAM-1 Levels in the Heart

**Figure 6:**
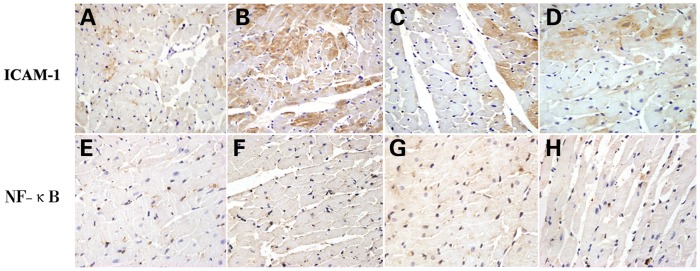
Immunohistochemistry staining of ICAM-1 and NF-κB p65 in the heart. A,E: NC group (n = 10); B,F: MS group (n = 10); C,G: MS+A group (n = 11); D,H: MS+H group (n = 11). The panels showed representative images of micrographs of immunohistochemistry staining in 4 groups. Original magnification, ×400.

The expression of NF-κB p65 and ICAM-1 in the rat hearts was evaluated by immunohistochemistry staining. In the MS rats, the expression of NF-κB p65 and ICAM-1 was significantly increased in the myocardial tissues compared to the NC group. However, both NF-κB p65 and ICAM-1 staining were decreased in either aspirin or HLJDT-treated rats compared to MS rats (Fig. 6).

### HLJDT Downregulates the Expression of Inflammatory Factors in the Heart

**Figure 7:**
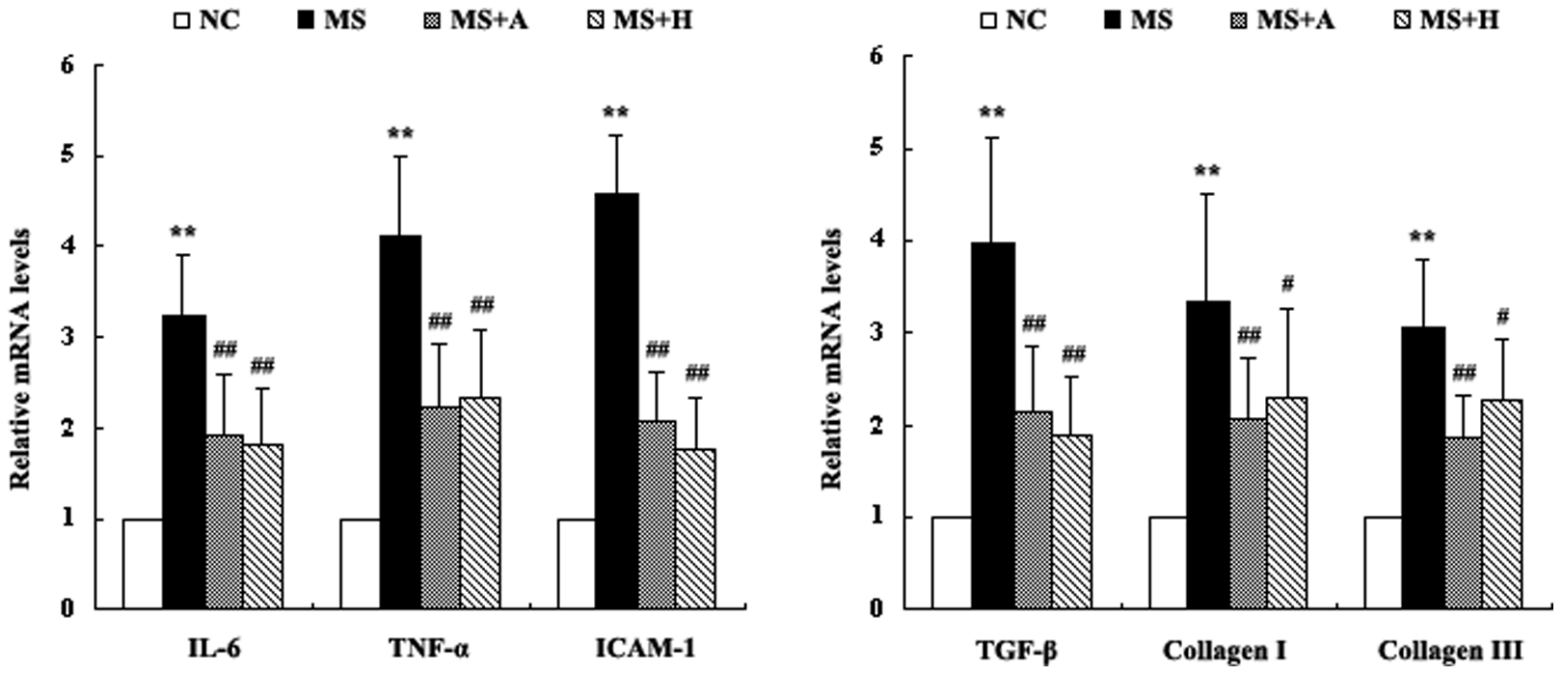
Expression of IL-6, TNF-α, ICAM-1, Collagen I/III and TGF-β1 mRNA in the heart. mRNA levels were detected by real-time PCR and each bar represented the mean ±SD. **P<0.01, *P<0.05 vs. NC; ^##^P<0.01, ^#^P<0.05 vs. MS. NC group (n = 10); MS group (n = 10); MS+A group (n = 11); MS+H group (n = 11).

The mRNA expression of IL-6, TNF-α, ICAM-1, collagen types I and III, TGF-β1and IKKβ were significantly increased in MS rats compared to NC rats (P<0.05, Fig. 7). However, aspirin and HLJDT significantly attenuated the increased expression of IL-6, TNF-α, ICAM-1, collagen I, collagen III and TGF-β1 mRNA (P<0.05, Fig. 7). The interclass analysis showed that these values were not significantly different between aspirin-treated and HLJDT-treated groups (P>0.05, Fig. 7).

### HLJDT Downregulates the Phosphorylation of IRS-1 in the Heart

**Figure 8:**
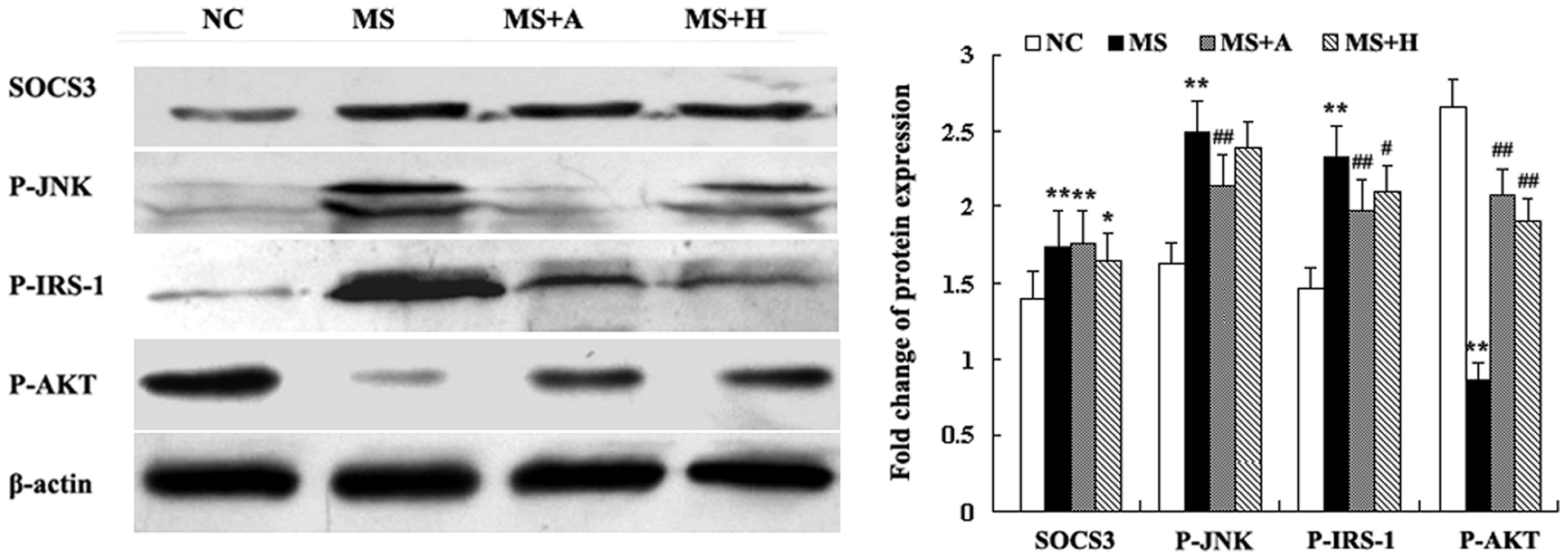
The activation of SOCS3, phospho-JNK, phospho- AKT and serine phospho- IRS1 in the heart. The left panels showed representative blots of the left ventricular. The right bar graph showed relative protein levels. Each bar represented the mean ±SD. **P<0.01, *P<0.05 vs. NC; ^##^P<0.01, ^#^P<0.05 vs. MS. NC group (n = 10); MS group (n = 10); MS+A group (n = 11); MS+H group (n = 11).

Western blot analysis revealed a profound up-regulation of the levels of SOCS3 and phospho-JNK by inflammation. At the same time, serine phospho-IRS1 was increased and phospho-AKT was decreased in the MS rats compared to the NC group (P<0.05, Fig. 8). Treatment with aspirin markedly attenuated the increases of phospho-JNK and phospho-IRS1 levels (P<0.05, Fig. 8), while the level of phospho-JNK was not affected by HLJDT treatment (P>0.05, Fig. 8). Moreover, compared with the MS rats, phospho-AKT level was increased in both aspirin and HLJDT-treated rats (P<0.05, Fig. 8).

## Discussion

In this study, we succeeded in producing obesity and metabolic disturbances similar to those seen in human MS by feeding obese-diet to rats. Based on this model we showed that MS caused significant myocardial lesions, increased the production of inflammatory cytokines and reduced insulin signaling in the heart. In previous study, we demonstrated that HLJDT treatment could inhibit inflammation and ameliorate MS-induced cardiac damage [15]. In the present study, we further investigated the mechanism by which HLJDT could improve IR, and compared the therapeutic effect between HLJDT and aspirin.

The obese-diet used in the present study was a high-fat, high-glucose and high-salt diet and sucrose solution, similar to the content of Western diets. We developed a MS model of rats with the following features: (1) Significant differences in BW between the obese-fed and normal-fed rats were developed from 20 weeks of age and persisted till 34 weeks. (2) Increased SBP was found in MS rats from 12 weeks of age, consistent with the classical features of MS [16]. (3) Low HDL-C was found in MS rats, while high TG and LDL-C were not found, suggesting that the lipid profile in MS rats is not exactly same as in human MS. (4) Elevated FBG, INS and IRI are regarded as a distinctive measure of IR. (5) The expression of inflammatory cytokines was increased and associated with increasing BW. These trends resembled those reported in human with obesity and IR.

MS is defined by the cluster of several classic cardiovascular risk factors (such as hypertension, obesity, type 2 diabetes, high triglycerides and low HDL-C), and the underlying pathophysiology is thought to be related to IR [17], [2]. In the present study, the obese-fed rats developed obesity with low HDL-C as well as elevated BP, plasma glucose and insulin, which are considered as MS-related features. Therefore, this animal model is regarded as a model that mimics human MS and could be used to investigate the pathophysiology of cardiac damage and develop novel pharmacotherapy and preventative measures for MS.

IR is defined as a state of reduced responsiveness to normal circulating levels of insulin and plays a major role in the development of MS [18], [2], [19], [20]. Thus we propose that IR is a key therapeutic target for the treatment and prevention of MS. Recent reports have linked the relation between obesity and IR in two different ways. First, ectopic lipid accumulation is a potential mechanism for this relationship. Second, a systemic chronic inflammatory response in obesity, characterized by altered cytokine production and activation of inflammatory signaling pathways, is another mechanism [21]. In a chronic inflammatory response of MS, inflammatory cytokines such as TNF-α, IL-1 and IL-6 were activated [4]. In addition, in adipose tissue of obese rodents and humans, TNF-α expression is increased [22], and reducing TNF-α expression could reduce IR in obese rodents [23].

Inflammation-mediated IR implicates several pathways including IKKβ/NF-κB, JNK and SOCS3, which are activated by inflammatory cytokines. Many studies demonstrated that inhibiting the activity of IKK, JNK and SOCS3 by pharmacological inhibition or gene knockout could improve IR [8], [24]–[27], [9]. In our study, the expression of inflammatory cytokines such as TNF-α, IL-1, and IL-6 was inhibited by pharmacological inhibitions, resulting in improving IR. Taken together, these results suggest that aberrant inflammation pathways may contribute to the onset of IR in MS rats.

Insulin signaling pathway in metabolism is regulated by the insulin-stimulated tyrosine phosphorylation of IRS-1 and the activity of PI3-kinase/AKT signaling pathway. The PI3K-dependent activation of the AKT kinase is known to control programmed cell death and cellular metabolism [28]. Moreover, activated AKT could preserve cardiac function because in rodent models of myocardial infarction, bone marrow–derived mesenchymal stem cells expressing constitutively activated AKT could enhance cardiomyocytes survival and organ function [29]. In our study, there were significant differences of left ventricle (LV) between the obese-fed and normal-fed rats, including LV structure (IVS and LVPW), diastolic function (A wave and E/A), and myocardial composition (the amount of collagen I/III). These differences were associated with reduced PI 3-kinase/AKT pathway. These results suggest that a long exposure to the obese-diet could inhibit insulin signaling, thereby inducing myocardial damage on both cardiac structure and function.

The efficacy of aspirin (acetylsalicylic acid) on reducing serine phosphorylation of IRS-1 associated with decreasing inflammatory cytokines has been reported [13]. In our study, by using aspirin as positive control we confirmed these findings. Thus we conclude that aspirin may enhance the insulin sensitivity by protecting IRS proteins from serine phosphorylation catalyzed by NF-κB and JNK.

HLJDT, a combination of herbs used in traditional Chinese medicine, consists of four medicinal compositions. Coptidis rhizome is the principal drug in HLJDT and Berberine hydrochloride, a key component of coptidis rhizome, has shown inhibitory effect on inflammation [30]. Although the effects of HLJDT on anti-inflammation and IR have been reported [31], [32], the underlying mechanism remains elusive. In our study, we found that HLJDT could reduce inflammatory mediators and improve IR, and this is associated with the inhibition of NF-κB activation induced by inflammatory cytokines. Compared with aspirin, the effects of HLJDT on reducing inflammation and increasing insulin-sensitivity were not significantly different.

In conclusion, insulin resistance induced by inflammation in MS could impair cardiac structure and function. Thus, increasing insulin sensitivity could contribute to MS therapy. The traditional Chinese medicine such as HLJDT could be an effective strategy to improve IR and protect MS patients from IR-mediated cardiac injury.
